# Associations between leadership styles and psychological distress among nurses

**DOI:** 10.1192/j.eurpsy.2025.1630

**Published:** 2025-08-26

**Authors:** N. Rmadi, A. Hrairi, F. Ben Atia, N. Kotti, F. Dhouib, M. Hajjaji, K. Jmal Hammami

**Affiliations:** 1 Occupational medicine department, Hedi chaker university hospital, University of Sfax; 2 Higher Institute of Nursing Sciences of Sfax, University of Sfax, Sfax, Tunisia

## Abstract

**Introduction:**

Leadership styles can either mitigate or exacerbate psychological distress, influencing job satisfaction, burnout, and overall well-being among nursing staff.

**Objectives:**

This study aims to explore how different leadership styles impact psychological distress in nursing professionals.

**Methods:**

We conducted a cross-sectional survey among nurses working in public hospitals and polyclinics in Sfax region. The questionnaire included socio-professional characteristics, assessment of leadership styles the Multifactor Leadership Questionnaire (MLQ) 6S and evaluation of nurses’ mental health using the Kessler Psychological Distress Scale 6 (K6).

**Results:**

A total of 200 physicians responded to the survey. The mean age was 33.24 ± 9.34 years with 70.3% being female. Mean scores of transformational, transactional and laissez faire styles were 25.6 ± 6.5, 12.8 ± 4.02 and 13.1 3.2 respectively. Psychological distress was likely to occur in 16.8% of the cases. Negative correlations were found between K6 score and transformational (p=0.00, r= - 0.81), transactional (p= 00, r=0.64) styles. However, laissez faire style was positively correlated with k6 score (p=0.024, r=0.16).

**Conclusions:**

By emphasizing transformational and transactional leadership styles, healthcare leaders can reduce psychological distress among nurses, enhancing their well-being and improving the overall effectiveness of healthcare system.

**Disclosure of Interest:**

None Declared

